# Minimally invasive procedure for removal of infected ventriculoatrial shunts

**DOI:** 10.1007/s00701-020-04675-1

**Published:** 2020-12-17

**Authors:** Lorenzo Magrassi, Gianluca Mezzini, Lorenzo Paolo Moramarco, Nicola Cionfoli, David Shepetowsky, Elena Seminari, Angela Di Matteo, Pietro Quaretti

**Affiliations:** 1 Neurosurgery, Department of Clinical, Surgical, Diagnostic and Pediatric Science, University of Pavia, IRCCS San Matteo Hospital Foundation, Neurochirurgia - Fondazione IRCCS Policlinico S. Matteo, V.le Golgi 19, 27100 Pavia, Italy; 2 Diagnostic Radiology, Interventional Radiology and Neuroradiology Unit, IRCCS Policlinico San Matteo Foundation, Pavia, Italy; 3 Unit of Infectious and Tropical Diseases, IRCCS Policlinico San Matteo Foundation, Pavia, Italy

**Keywords:** Ventriculoatrial shunt, Hydrocephalus, Endocarditis, Right atrium, Endovascular removal

## Abstract

**Background:**

Ventriculoatrial shunts were one of the most common treatments of hydrocephalus in pediatric and adult patients up to about 40 years ago. Thereafter, due to the widespread recognition of the severe cardiac and renal complications associated with ventriculoatrial shunts, they are almost exclusively implanted when other techniques fail. However, late infection or atrial thrombi of previously implanted shunts require removal of the atrial catheter several decades after implantation. Techniques derived from management of central venous access catheters can avoid cardiothoracic surgery in such instances.

**Methods:**

We retrospectively investigated all the patients requiring removal of a VA shunt for complications treated in the last 5 years in our institution.

**Results:**

We identified two patients that were implanted 28 and 40 years earlier. Both developed endocarditis with a large atrial thrombus and were successfully treated endovascularly. The successful percutaneous removal was achieved by applying, for the first time in this setting, the endoluminal dilation technique as proposed by Hong. After ventriculoatrial shunt removal and its substitution with an external drainage, both patients where successfully weaned from the need for a shunt and their infection resolved.

**Conclusion:**

Patients carrying a ventriculoatrial shunt are now rarely seen and awareness of long-term ventriculoatrial shunt complications is decreasing. However, these complications must be recognized and treated by shunt removal. Endovascular techniques are appropriate even in the presence of overt endocarditis, atrial thrombi, and tight adherence to the endocardial wall. Moreover, weaning from shunt dependence is possible even decades after shunting.

**Supplementary Information:**

The online version contains supplementary material available at 10.1007/s00701-020-04675-1.

## Introduction

In pediatric patients, ventriculoatrial shunts (VAS) are now rarely implanted as a first-line treatment of hydrocephalus and their use is limited to a second-line treatment when endoscopic third ventriculostomy (ETV) is not indicated or has repeatedly failed, and the peritoneum becomes an unsuitable site for shunting [10]. The main reasons for downgrading VAS to second-line treatment for hydrocephalus are its documented long-term severe cardiac and renal complications. Chronic atrial perforation [13], recurrent endocarditis [5], immune-complex glomerulonephritis [3], and tricuspid incompetence [31] have all been associated with VASs. Nevertheless, in elderly patients affected by normal pressure hydrocephalus, some have recently advocated VASs as an alternative primary treatment option to venticuloperitoneal shunts (VPSs) because, according to these authors, VASs are less likely to undergo obstruction and require shunt revision [19, 25, 28]. The apparent differences in the incidence of complications among pediatric and elderly patients may be explained by the differences in the length of follow-up of the studies in the two age groups. Follow-up in the elderly was limited to few years with a median ranging in those studies from 15 [19] to 42 [28] months, while in pediatric patients reported follow-ups were typically well above 10 years [8, 26].

Strong support for early recognition and treatment of VAS complications come from the documented reversibility of most complications after removal of the VAS especially if they result from bacterial infection [8, 39]. Removal of a chronically implanted VAS catheter from the right atrium is usually not straightforward due to the frequent presence of adhesions to the endocardial structures [4] and/or the presence of a large atrial thrombus [38].

Thoracotomy is considered the method of choice for removal of a VAS when perforation of the atrial wall [13, 27] or a large thrombus is present [35], while less invasive endovascular techniques are more commonly employed for removal of a broken VAS catheter [12, 36].

We now report the endovascular removal of two infected VAS catheters associated with large thrombi that were implanted 28 and 40 years before and were both tightly adherent to the endovascular and endocardial wall. After removal of the catheters, both patients were progressively weaned from the intracerebral shunts, and their cardiac problems resolved.

## Methods and materials

### Patients

The study period was August 2015 through August 2020. All adult patients admitted at the Fondazione I.R.C.C.S. Policlinico San Matteo, Pavia with a complication linked to VAS were reviewed. Timing and approach of surgical intervention were determined on a case-by-case basis by the attending neurosurgeon and the interventional radiologist in collaboration with an infectious disease specialist. All patients signed an informed consent authorizing the use of their anonymized clinical data for retrospective analysis and clinical research.

### Statistics

The working period of VAS was calculated by using the interval between VAS implantation and explantation reported in each paper and the equivalent period for our two patients. These data were analyzed according to the Kaplan–Meier model, and significant differences in time to explantation of AVS were evaluated by the log-rank test by using MedCalc software, version 18.2.1. (MedCalc Software bv, Ostend, Belgium; https://www.medcalc.org) A *p* value < 0.05 was considered statistically significant.

## Results

In the study period encompassing 5 years, two adult patients underwent shunt removal for VAS complications. Their clinical history is summarized below:

### Patient no. 1

**Fig. 1:**
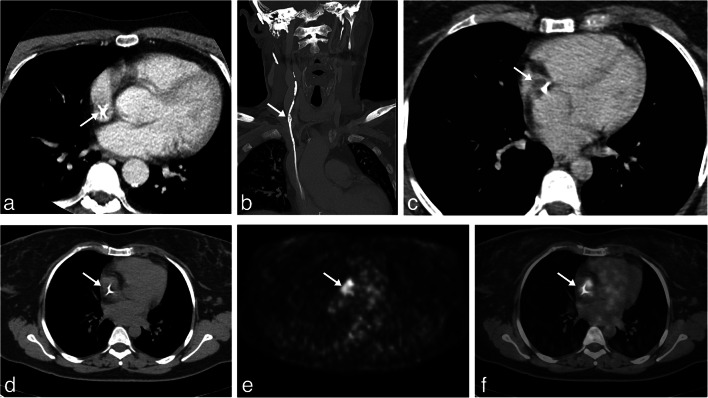
Atrial condition before VAS removal. **a** Preoperative thoracic axial CT scan with contrast at the level of the atrium showing the VAS catheter (arrow) in patient 1. **b** Preoperative coronal reconstruction of the trajectory of the VAS catheter in patient 1 in the internal jugular vein, superior vena cava and atrium, radiolucency of the catheter was increased by calcium deposition in the reactive tissue surrounding the catheter, the arrow indicates the presence of macroscopic calcifications around the catheter. **c** Preoperative thoracic axial CT scan with contrast at the level of the atrium showing the VAS catheter in patient 2. The arrow indicates a large atrial thrombus associated with the catheter. **d** Axial thoracic CT scan of patient 2, the arrow indicates the VA catheter inside the right atrium. **e** [^18^F] fluoro-2-deoxy-d-glucose (FDG) PET scan image corresponding to thoracic CT scan in 1d, FDG accumulation (SUV 7.4) in close association with the position of the VAS-catheter (arrow) is visible. **f** Composite image demonstrating the colocalization of the FDG accumulation with the thrombus and reactive tissue surrounding the atrial catheter (arrow)

**Fig. 2:**
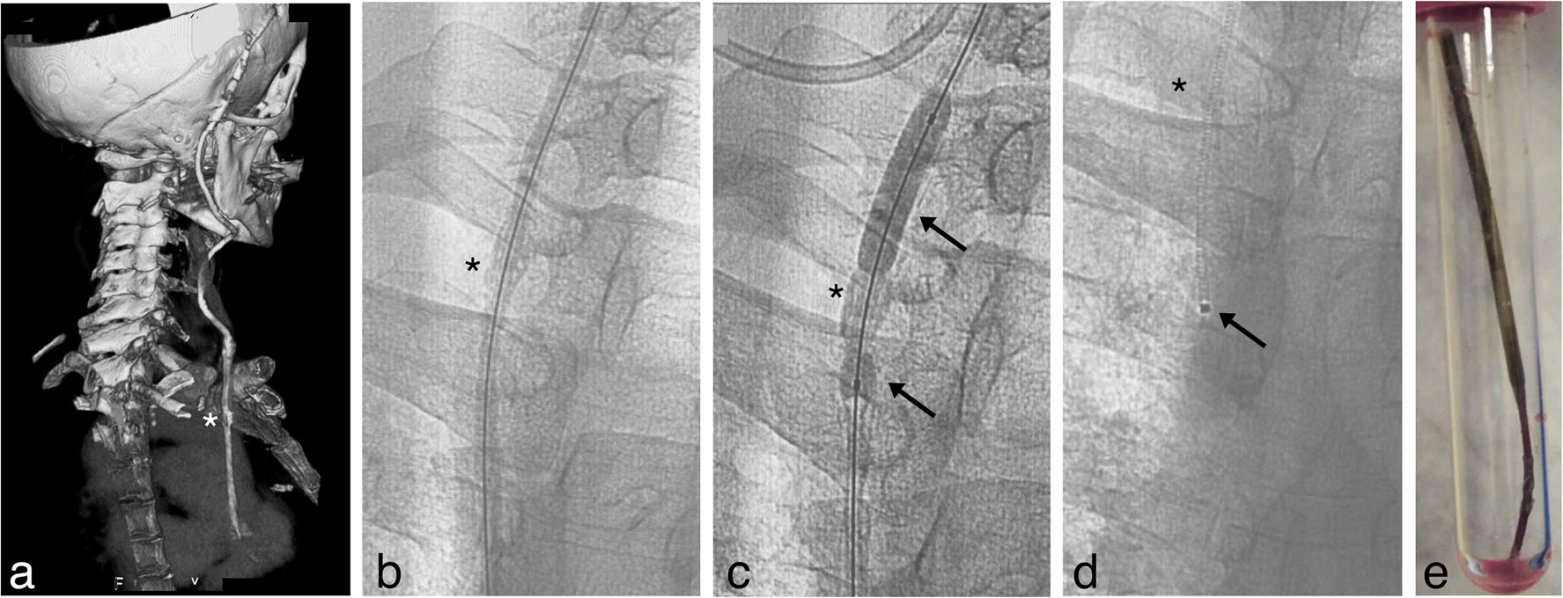
Removal of VAS catheter in patient 1. **a** Preoperative 3D CT scan reconstruction of the VAS catheter path from the valve to the right atrium, white asterisk (*) marks the site of macroscopic calcifications surrounding the catheter. **b** Fluoroscopy antero-posterior projection, the 0,018 inch guidewire is visible inside the VAS catheter, black asterisk (*) marks the site of the same macroscopic calcification shown in Fig. 1a. **c** Fluoroscopy antero-posterior projection, the VAS catheter was dilated by a 6 mm diameter Sterling balloon catheter. The calcification surrounding the VAS catheter partially restricts the dilation of the lumen compared to the adjacent segments (arrows), asterisk as in Fig. 1b. **d** Fluoroscopy antero-posterior projection, obtained after complete removal of the VAS catheter arrow points to an angiography catheter introduced after the VAS catheter was completely removed. **e** Picture of the distal portion of the VAS catheter immediately after extraction, the catheter is encrusted by calcified reactive tissue and partially deformed

A 59-year-old male had undergone implantation of a cystoatrial shunt for treatment of an arachnoid cyst of the right frontal lobe in 1976; no revision of the implant was ever performed. In 2014, he underwent kidney transplantation for end stage polycystic kidney disease. After transplantation, the patient presented with recurrent urosepsis. In 2016, during a mild septic episode, a floating atrial thrombus associated to the VAS catheter tip was revealed by transoesophageal echocardiography (TEE). Cerebrospinal fluid (CSF) collected by direct puncture of the valve of the VAS was sterile, and biochemical and cellular parameters were normal. During 4 weeks of antibiotic and low molecular weight heparin treatment repeated examinations by (TEE) demonstrated a progressive reduction in the size of the thrombus until it completely disappeared. One month after suspension of antibiotic therapy, the patient was readmitted to the hospital with a new mild septic episode, a methycillin resistant *S. aureus* was isolated in the hemocultures, and an atrial thrombus associated with the catheter tip was again visible at TEE. Considering failure of conservative treatment, we decided to remove the atrial catheter (Fig. 1a), we exposed the VAS catheter at its entrance into the venous system distal to the valve at the neck, and after cutting the catheter, its proximal stump was connected to an external ventricular drainage (EVD), the intravascular portion of the catheter was anchored to the wall of the superior vena cava where pericatheter calcifications were demonstrated by CT scan (Fig. 1b) and could not be removed by simple manual traction. Applying a refinement of the endoluminal dilation technique [33] as pioneered by Hong [18] the catheter was uneventfully removed [33] (Fig. 2). Briefly: we inserted a 4Fr valved sheath (Terumo) through the catheter exposed at the cervical level, then we advanced a 0.018 guidewire through the lumen of the catheter and navigated in the inferior vena cava, after performing several dilations of the catheter with a 6 diameter Sterling balloon catheter (Boston SC), leaving the guidewire in place, we again pulled on the atrial catheter that broke at the level of the calcifications. After removal of the segment of the catheter proximal to the calcifications, we captured from femoral approach with a 30-mm snare (Goose Neck, Bard) both the distal end of the guidewire and the distal segment of the catheter and completely removed it through a combined endovascular access through the internal jugular and femoral veins. After the procedure, the patient was progressively weaned from the EVD which was then closed 2 weeks after surgery and completely removed 5 days after closure of the EVD. The neurological examination of the patient remained normal and repeated CT scans did not show any relapse of the arachnoid cyst (Fig. 4a, b). Four years after VAS removal, the neurological condition of the patient remains stable without new septic episodes.

### Patient no. 2

A 48-year-old female affected by myelomeningocele and hydrocephalus was operated shortly after birth and after closing the spinal defect, a VPS was implanted. In the following years, the shunt required three revisions until in 1992 the tip of the peritoneal catheter was extruded from the anus after spontaneous rectal perforation. After removal of the VPS, a VAS was implanted; the ventricular catheter and the valve of the VAS were changed in 2013 after transitory CSF infection but the atrial catheter, which was properly functioning in the absence of intracardiac vegetations or thrombi visible on TEE, was left in place.

In November 2019, after relapsing episodes of fever, she was admitted to the infectious disease unit after demonstrating a large thrombus associated with the distal tip of the VAS catheter by TEE. Multiple cultures of blood and CSF resulted sterile and an *Escherichia coli* was isolated from the urine. She started antibiotic therapy and low molecular weight heparin, with thrombus reduction. In March 2020, she was readmitted to the Hospital for relapsing fever; Cutibacterium acnes were isolated from multiple blood cultures, and signs of right heart failure were evident. A large thrombus around the tip of the atrial catheter was again demonstrated by TEE and thoracic angioCT (Fig. 1c). A [^18^F] fluoro-2-deoxy-d-glucose (FDG) PET scan showed enhanced uptake of the tracer with a standardized uptake value (SUV) of 7.4 at the level of the thrombus (Fig. 1d, e, f). The VAS was considered as the source of the relapsing infection, and, after a collegial discussion, the removal of the whole system was planned.

**Fig. 3:**
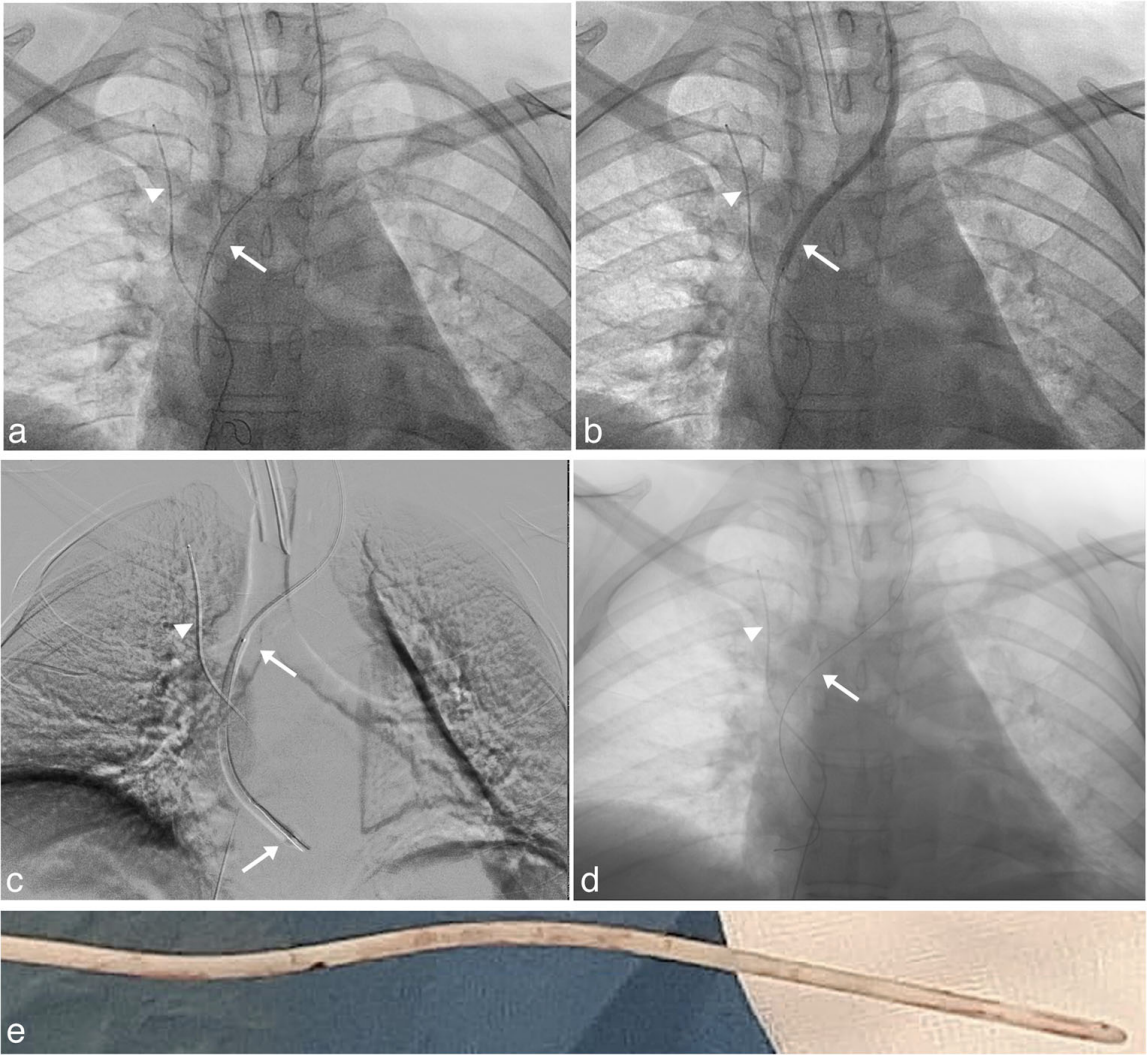
Removal of VAS catheter in patient 2. **a** Fluoroscopy antero-posterior projection, a 0.018 inch guidewire is visible inside the atrial catheter (arrow), a safety wire introduced through the femoral vein (arrowhead) was placed as a guard. **b** Fluoroscopy antero-posterior projection, the VAS catheter was dilated by a 3.5-mm diameter Amphirion balloon catheter. Arrow and arrowhead as in Fig. 3a. **c** Digital subtraction antero-posterior radiogram, the VAS catheter was distally dilated by a 3.5-mm diameter Amphirion balloon catheter reaching the distal end of the VAS catheter. Arrow and arrowhead as in Fig. 3a. **d** Fluoroscopy antero-posterior projection, obtained after complete removal of the VAS catheter with the safety wire (arrowhead) and guidewire still in place (arrow). **e** Picture of the distal portion of the VAS catheter obtained immediately after extraction, the catheter is almost completely clean from thrombotic material that was present in vivo

**Fig. 4:**
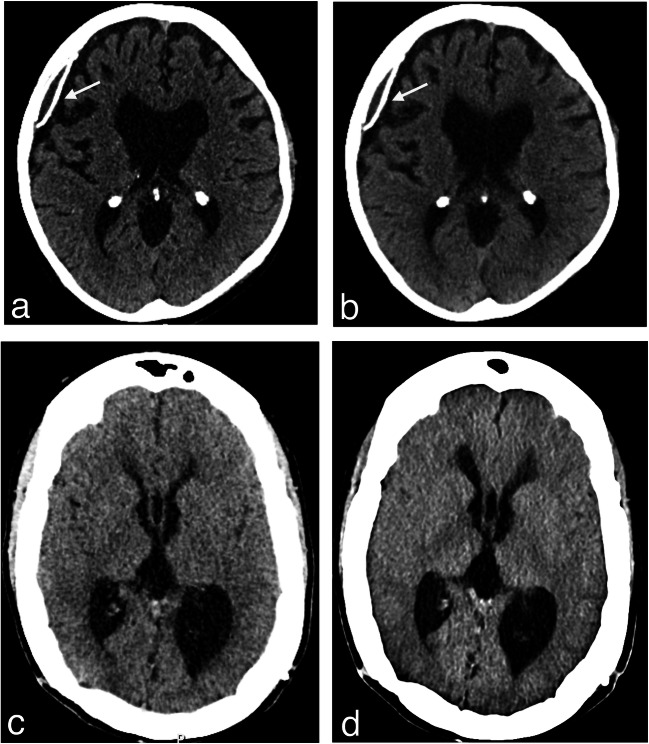
Stability of ventricular system after VAS inactivation. CT scans obtained from patient 1 (**a** and **b**) and patient 2 (**c** and **d**) before surgery (**a** and **c**) and after removal of the VAS catheter from the atrium and definitive inactivation of the shunts (**b** and **d**): no appreciable increase in the diameter of the cyst and ventricles is visible. Arrows in Fig. 4a and b point to the calcified wall of the cyst

Under general anesthesia (upon request of the patient), we exposed the VAS catheter at its entrance into the venous system in the neck distal to the valve and, after cutting the catheter, we connected its proximal stump to an EVD. As expected, the intravascular portion of the catheter was adherent to the vessel walls and endocardium and could not be removed by simple manual traction. Notably, the distal end of the atrial catheter appeared medialized and not free floating in the atrium on fluoroscopy. As described for patient no. 1, a 4F introducer Terumo was inserted over a 0.014 inch guidewire until the distal end of catheter was reached. Then, cautiously an over-the-wire 3.5 × 80 mm balloon catheter Amphirion (Medtronic) was advanced on the guide wire and dilated two times inside the catheter (Supplementary video 1). After that, keeping in site the guidewire the catheter was easily retired outside (Fig. 3). Pulmonary arterial pressure was within normal limits. Femoral access previously gained for any potential rescue manoeuvre was unutilized. After the procedure, the patient was progressively weaned from the EVD which was closed 2 weeks after surgery and removed 6 days after closure. During this surgery, the ventricular catheter was ligated and left in place due to its tight adherence that made removal by manual traction hazardous. The neurological examination of the patient remains stable and repeated CT scans excluded any progressive enlargement of the ventricles (Fig. 4c, d).

Supplementary Video 1Patient 2, digital angiography showing advancement and dilation inside the AVS catheter of a 3.5 × 80 mm balloon Amphirion (Medtronic). (M4V 2174 kb)

## Discussion

**Fig. 5:**
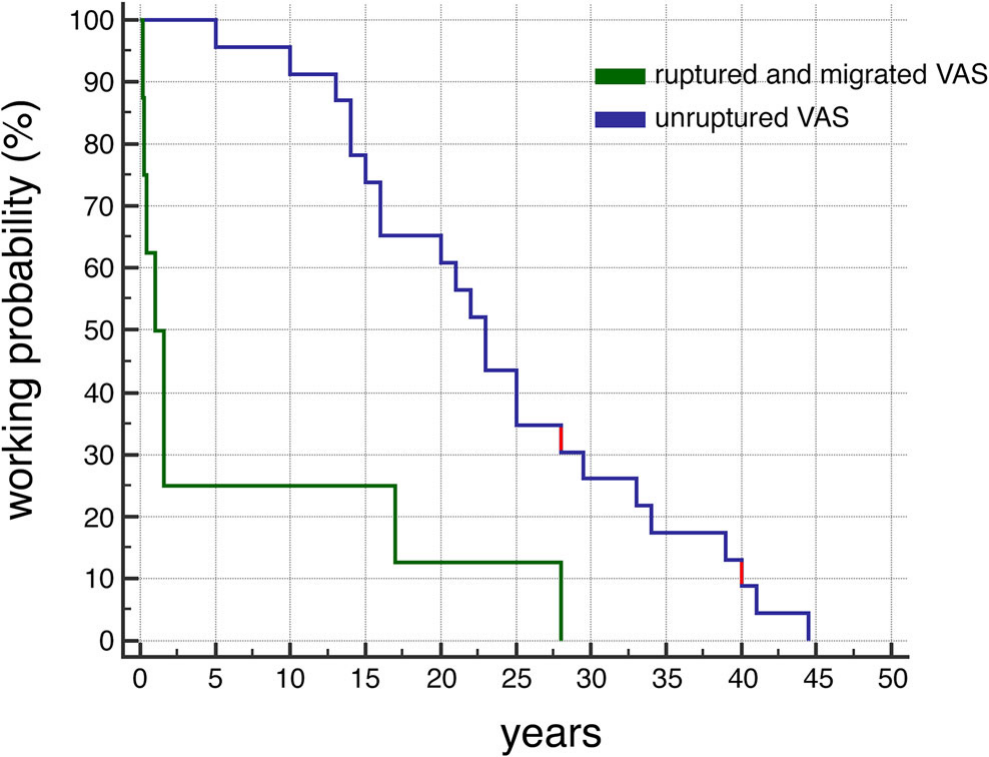
Plot of the probability of working VAS with time. Data for plot were derived from the literature together with the present cases (red). Median lag before VAS removal for thrombus formation and/or infection was 23 years (min 5 years, max 44.5 years, *n* = 23 patients) while median lag for rupture and or migration of the atrial catheter was 1.29 years (min 0.16 years, max 28 years, *n* = 8 patients)

Patients carrying a VAS are becoming less and less frequent since this modality of treatment was relegated to a second line treatment for hydrocephalus. Nevertheless severe cardiac, renal [3, 39], and hepatic [34] complications of VASs may arise decades after the initial treatment. We presented two cases of infected atrial VASs developing severe cardiac complications more than 25 years after VA shunt implantation. We found several reports describing complications of VAS in adults occurring after the postoperative period (1 month after shunt implantation). Analysis of the reported late complications requiring AVS removal together with the two described in the present work, shows that complications associated with rupture and displacement of the atrial catheter [2, 6, 15, 20, 27, 36, 40] occur significantly earlier than complications not associated to rupture or displacement of the catheter [1, 4, 5, 7–9, 11, 16, 29, 30, 32, 34, 38, 39] (Fig. 5). We hypothesize that this difference reflects the common tendency of atrial catheters to become adherent to vascular and endocardial structures thus decreasing the possibility of displacement with time, but further studies are necessary to clarify this point.

In both our patients, despite tight adhesion of the shunt catheter to the superior vena cava (patient 1) or atrium (patient 2) and the presence of large atrial thrombotic vegetations, we were able to remove the catheters endovascularly without complications and with complete reversal of the associated signs and symptoms (right heart failure).

Retrieval of broken ventriculoatrial catheters trough an endovascular approach has a long history starting with open approaches requiring exposure of the involved jugular vein [37] and gradually developing into a full endovascular technique [12, 20]. Endovascular approaches were mainly used for retrieval of broken or displaced atrial catheters. However, more complicated cases where the catheter is infected, or enclosed by a large thrombus, or adherent to the atrial wall or to the leaflets of the tricuspid valve are still treated by thoracotomy [13, 16, 27]. Moreover, emergency thoracotomy is still considered mandatory after cardiac tamponade due to accumulation of CSF in the pericardium secondary to chronic perforation of the atrium by the tip of a VAS catheter which then protrudes into the pericardial space [13, 22, 27]. Finally, atrial catheters associated with tricuspid regurgitation if paucisymptomatic are sometimes left in place with strict clinical monitoring [31].

The first of our patients developed infectious endocarditis few months after kidney transplantation for end stage kidney disease due to polycystic kidney disease. Immune-complex membranoproliferative glomerulonephritis has also been described as a complication of chronic bacterial infection of VASs [3, 39] that can lead to kidney failure and kidney transplantation. VAS removal is usually followed by complete regression of the glomerulonephritis [3], and it is thus mandatory whenever possible.

Another severe complication of VASs is development of pulmonary hypertension secondary to recurrent pulmonary embolization due to chronic formation of thrombi in and around the atrial catheter of the shunt [14, 30]. The incidence of this complication in a large case series with a median interval between shunt insertion and diagnosis of 15 years was 8% (3 of 38 patients) [23] Moreover, risk of developing pulmonary hypertension following VAS increases after shunt infection [2, 7, 11].

In both our patients before removing the catheters, we tried conservative treatment with antibiotics and heparin [9]. However, despite an initial improvement, the infection and endocardial vegetations recurred shortly after stopping the antibiotic treatment. Removal of the atrial catheter in our cases was necessary for infection eradication with immediate resolution of all endocardial thrombi. Despite tight adhesion of the catheters to endovascular structures and presence of thrombi, we were able to remove the catheters through a novel endovascular approach. We applied for the first time, to the best of our knowledge, the endovascular catheter dilation technique introduced in the last decade for removal of adherent dialytic central venous catheters to very old atrial catheters carried by our patients. As suggested by Hong, the endocatheter dilation by inflation of low-profile balloon catheters permitted to disrupt fibrous encasement around the distal VAS catheter, allowing the outside retrieval. This technique was successful despite the presence of calcifications encasing the catheter in our first patient and tight adherence to the atrial wall in the second patient. The endocatheter dilation technique [33] we adopted in our patients resulted as efficient as the laser-assisted extraction technique that has been used in the past to remove an entrapped VAS catheter [4] with the advantage of not requiring an endovascular laser apparatus that has a significant initial cost and is not widely available.

Both our patients were made shunt free despite a long story of shunt dependence and preoperative evidence that at least the proximal catheter was patent since in both patients we collected ventricular CSF by tapping the valve before removal of the VAS, and in both CSF was regularly drained by the EVD while it was progressively raised before we closed the system. One patient originally, had the shunt implanted to drain an arachnoid cyst into the atrium, leading to an initial reduction and later stabilization of the cyst. The cyst did not recur after removal of the shunt. The other patient carrying a left VAS for hydrocephalus eleven years before removal, required emergency surgery to replace the malfunctioning valve and ventricular catheter after acutely developing obvious sign of intracranial hypertension including headache, blurred vision followed by coma. Despite this, both patients have by now long surpassed their 1 month follow-up which is considered the typical period for developing clinical symptoms after failure of substituting a ventricular shunt by ETV [17]. Although, the number of patients in our series is too limited for any conclusion regarding shunt dependence, and examples of shunt dependence after almost five decades have been reported [21] we cannot help but notice that older patients with a long history of shunting are also better candidates for successful ETV [17, 24] being less shunt dependent than patients with a similar initial indication for shunting but a shorter history of shunting.

## Conclusion

VASs are rarely implanted nowadays, but many patients still carry this type of device. Neurosurgeons should not forget that the severe complications linked to VAS may present even decades after shunt implantation. In our experience, endovascular removal of the atrial catheter through the endoluminal dilation technique seems to be safe even in the presence of large thrombi and tight adhesion to the endocardial structures. In the absence of cardiac perforation or severe pulmonary hypertension, the endovascular approach should be tried before referring the patients to the cardiac surgeon. Finally, a short period of conversion to external drainage of the shunt may be useful not only in order to monitor sterility of the CSF during antibiotic therapy but also to test whether definitive weaning from any type of shunt is possible.
